# PGRMC1 and PAQR4 are promising molecular targets for a rare subtype of ovarian cancer

**DOI:** 10.1515/biol-2022-0982

**Published:** 2024-10-26

**Authors:** Kamila Kozłowska-Tomczyk, Norbert Borski, Paulina Głód, Justyna Gogola-Mruk, Anna Ptak

**Affiliations:** Laboratory of Physiology and Toxicology of Reproduction, Institute of Zoology and Biomedical Research, Jagiellonian University, Gronostajowa 9, 30-387, Krakow, Poland; Doctoral School of Exact and Natural Sciences, Faculty of Biology, Institute of Zoology and Biomedical Sciences, Jagiellonian University, Gronostajowa 9, 30-387, Krakow, Poland

**Keywords:** ovarian cancer, cell lines, ZIP9, OXER1, mPR, PGRMC1, bioinformatics, prognosis

## Abstract

The heterogeneity of ovarian cancer (OC) has made developing effective treatments difficult. Nowadays, hormone therapy plays a growing role in the treatment of OC; however, hormone modulators have had only limited success so far. To provide a more rigorous foundation for hormonal therapy for different OC subtypes, the current study used a series of bioinformatics approaches to analyse the expression profiles of genes encoding membrane progesterone (PGRMC1, progestins and the adipoQ receptor [PAQR] family), and androgen (zinc transporter member 9 [ZIP9], OXER1) receptors. Our work investigated also their prognostic value in the context of OC. We found differences in expression of ZIP9 and OXER1 between different OC subtypes, as well as between patient tumour and normal tissues. Expression of mRNA encoding PAQR7 and PAQR8 in a panel of OC cell lines was below the qPCR detection limit and was downregulated in tumour tissue samples, whereas high expression of PGRMC1 and PAQR4 mRNA was observed in rare subtypes of OC cell lines. In addition, chemical inhibition of PGRMC1 reduced the viability of rare OCs represented by COV434 cells. In conclusion, PGRMC1 and PAQR4 are promising targets for anticancer therapy, particularly for rare subtypes of OC. These findings may reflect differences in the observed responses of various OC subtypes to hormone therapy.

## 1 Introduction

Ovarian cancer (OC) is one of the top five causes of death from gynaecological cancers in developed countries [1]. The biggest problem in the case of OC is detection at a late stage due to the aggressive behaviour of the tumour and a lack of effective early diagnostic tests. Consequently, survival rates are low (5-year survival = 10–30%) [2]. In 2020, approximately 21,750 cases were diagnosed in the United States, and 13,940 women died [3]. Despite testing various treatment strategies and new cytotoxic agents, the optimal first-line therapy and survival rates have not changed significantly since the introduction of platinum and taxane agents [4,5,6]. Given this situation, there is an urgent need to find biomarkers of prognosis and response to therapeutic intervention, as well as novel treatment strategies.

OC comprises many types, as well as a number of rare subtypes, each with different cells of origin and biology; therefore, ovarian tumours require various treatment approaches. Epithelial OC (EOC) accounts for 90% of all diagnosed subtypes and includes all cancers arising from the epithelium and involving the ovary [7]. The most common histological types are high-grade serous (HGS), low-grade serous (LGS), endometrioid, clear cell, and mucinous. Extremely rare OCs include small cell carcinomas of the ovary, which are a group constituting <1% of total cases. Apart from typical OC, there are also distinguished sex cord-stroma tumours, which arise from the sex cord (e.g. granulosa cell tumours [GCT]) or stromal cells (or both). These tumours often secrete steroid hormones, particularly androgens or oestrogens, and account for approximately 7% of all ovarian malignancies [8]. Therefore, the main problem in OC research is disease heterogeneity. To take this heterogeneity into consideration during this study, we used cell lines representative of EOC (SK-OV-3, OVCAR-3, with HOSEpiC as a non-cancerous control) and GCT (KGN, COV434, with HGrC1 as a non-tumourous control). The SK-OV-3 cell line is a classified LGS or clear cell cancer, whereas OVCAR-3 is representative of HGS OC and is the only common OC type used in the current study. Recent studies suggest that COV434 cells are representative of small cell carcinomas of the ovary rather than GCT [9,10,11].

The exact causes of OC remain unclear, although several risk factors have been identified, including early menarche and late menopause, nulliparity, obesity, age at menopause, hormone replacement therapy during menopause, and ethnicity [12]. Most of these risk factors are associated with changes in the levels of sex hormones (oestrogens, androgens, and progestins) during a woman’s lifetime. Sex hormones regulate expression of genes in the nucleus by binding to cognate nuclear receptors. Previous studies show that OC is not generally considered to be oestrogen-sensitive, whereas androgens promote progression of OC and progesterone may be a protective factor [13].

A number of studies report that expression of nuclear receptors for sex hormones is positively associated with OC prognosis or treatment [12,14–18]; however, these studies are controversial. Recent data suggest that sex hormones also exert non-genomic functions by binding at or near to the plasma membrane to induce rapid changes in cell physiology. Molecular mediators of androgenic action include the plasma membrane receptors zinc transporter member 9 (ZIP9) and oxoeicosanoid receptor 1 (OXER1) [19]. Furthermore, non-classical progesterone receptors involved in extranuclear signalling are classified into two groups: the class II progestins and the adipoQ receptor (PAQR) family (also called mPRs) and the b5-like haem/steroid binding protein family (also called MAPRs) [19].

The ability to bind hormones and hormone-like substances, and to activate non-genomic pathways that play a pivotal role in cancer progression, makes membrane steroid receptors interesting and potential new drug targets. Therefore, we conducted PCR and bioinformatics analyses to investigate the expression profile, mutation status, and prognostic value of membrane androgen and progesterone receptors ZIP9, OXER1, PAQR7, PAQR8, PGRMC1, or PAQR4 and identified factors prognostic for OC. In addition, we analysed the expression profile of membrane sex steroid receptors in human non-cancer and cancer cell lines representing different subtypes of human OC. Finally, we investigated whether the chemical inhibition of PGRMC1 affects the viability of a human rare OC cell line.

## 2 Materials and methods

### 2.1 Cell culture

**Figure 1:**
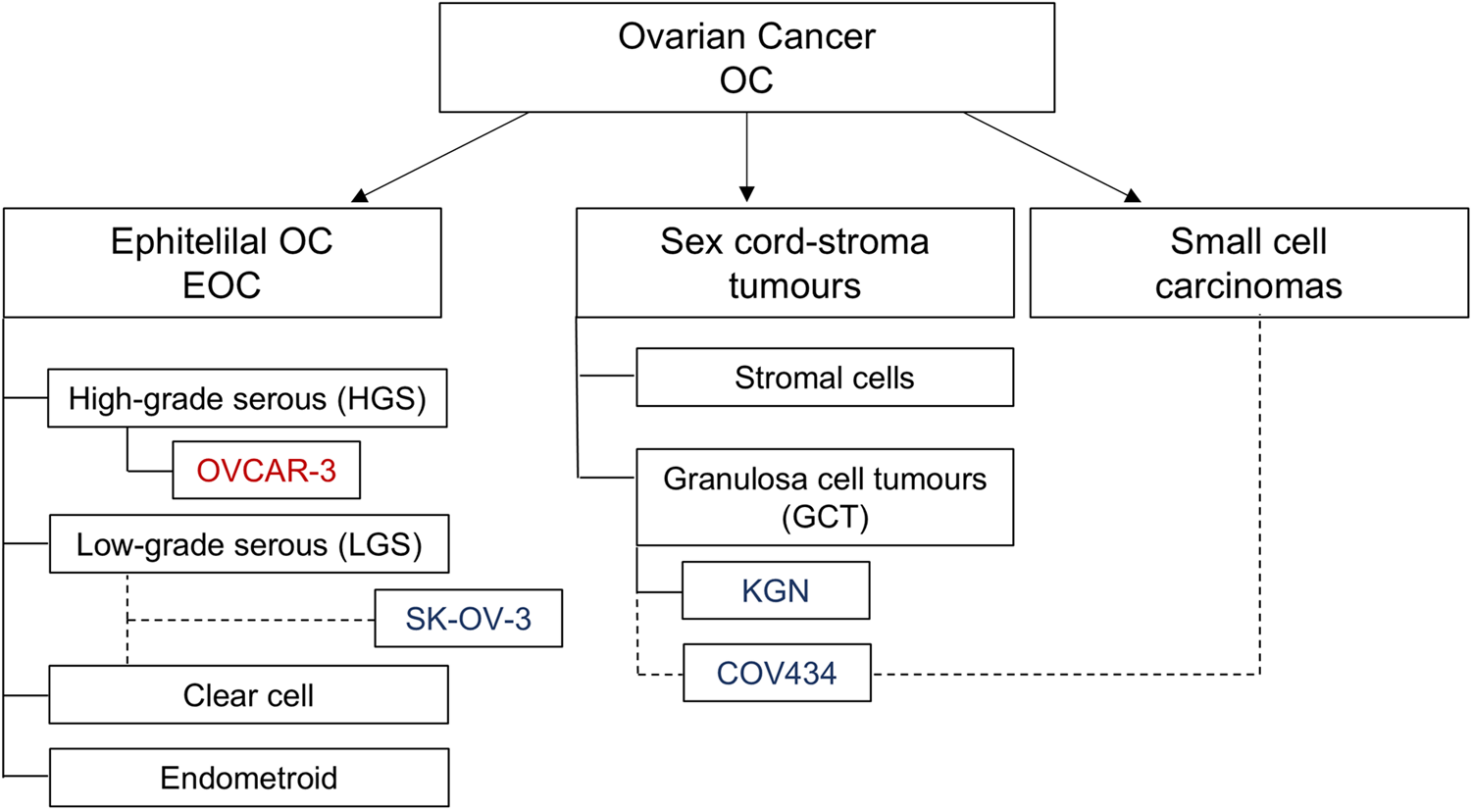
Schematic presentation of the ovarian cancer subtypes classification including tested cancer cell lines. The cell line representing a common type is marked in red, and those representing rare types are in blue.

Four human OC cell lines were used as an *in vitro* model: the human epithelial ovarian carcinoma cell line OVCAR-3 (American Type Culture Collection; Manassas, VA, USA, Catalog No. HTB-161, which is representative of HGS OC); the SK-OV-3 cell line (European Collection of Authenticated Cell Cultures [ECACC]; Sigma-Aldrich, St. Louis, MO, USA Catalog No. 91091004, which is a representative clear cell cancer or LGS [10,11]); the human granulosa cell tumour-derived cell line KGN (RBRC-RCB1154, Riken Cell Bank, Ibaraki, Japan; Catalog No. RBRC-RCB1154, obtained with approval from Drs. Yoshiro Nishi and Toshihiko Yanase); and COV434 cells (ECACC; Sigma-Aldrich, Catalog No. 07071909), which were recently suggested to represent small cell carcinoma of the ovary (a hypercalcaemic OC type) rather than a GCT [9]. The human ovarian surface epithelial cell line HOSEpiC (ScienCell Research Laboratories, Carlsbad, CA, Catalog No. 7310) and the human non-luteinizing granulosa cell line HGrC1 (a kind gift from Dr. Ikara Iwase; Nagoya University, Japan) were used as non-cancer controls (Figure 1).

OVCAR-3 and HOSEpiC cells were cultured in phenol red-free RPMI 1640 (ThermoFisher Scientific, Waltham, MA, USA). SK-OV-3 cells were cultured in McCoy’s 5A medium (Sigma-Aldrich, St. Louis, MO, USA). KGN cells were cultured in phenol red-free Dulbecco’s modified Eagle’s medium (DMEM)/HAM’s F12 (ThermoFisher Scientific). COV434 and HGrC1 cells were maintained in DMEM (Sigma-Aldrich, St. Louis, MO, USA) without phenol red and supplemented with 2 mM l-glutamine. All media were supplemented with 10% heat-inactivated, charcoal-stripped foetal bovine serum (Biowest, Nuaillé, France). All cells were maintained in humidified incubator (37°C, 95% air, 5% CO_2_).

### 2.2 RT-qPCR

Expression of genes by HGrC1, HOSEpiC, OVCAR-3, SK-OV-3, KGN, and COV434 cells was measured by real-time qPCR. First, cells from each cell line were seeded into six-well plates and cultured for 72 h. *PGRMC1* expression following treatment with AG-205 was analysed after 24 h exposure in COV434 cells. After this time, total RNA was extracted from the same amount of cells, and cDNA synthesis was performed using the TaqMan Gene Expression Cells-to-CT kit (Applied Biosystems/ThermoFisher Scientific). The lysis solution contained DNase I to remove contaminating genomic DNA. Cell lysates were reverse transcribed to synthesise cDNA using a convenient RT Enzyme Mix and RT Buffer. RT-qPCR was performed using the StepOnePlus real-time PCR system (Applied Biosystems/ThermoFisher Scientific) in a 96-well optical plate; each well contained 20 µl of reaction mix comprising RNA, TaqMan gene expression assay and TaqMan gene expression master mix with ROX passive reference dye (Applied Biosystems/ThermoFisher Scientific). The thermal cycling conditions were as follows: 50°C for 2 min, 95°C for 10 min, and then 40 cycles of 95°C for 15 s and 60°C for 60 s. The following TaqMan gene expression assays were used: *ZIP-9* (SLC39A9; Hs04276955_m1); *OXER1* (OXER1; Hs00536961_s1); *PGRMC1* (PGRMC1; Hs00998344_m1); *PAQR4* (PAQR4; Hs00373823_m1); *PAQR7* (PAQR7; Hs01372781_m1); and *PAQR8* (PAQR8; Hs00370233_m1). Expression was normalised to that of *GAPDH* (4310884E). Relative expression was quantified using the 2^−ΔΔCt^ method [20].

### 2.3 Analysis of the gene expression profiling interactive analysis (GEPIA)

The GEPIA data set is a web-based tool used to deliver fast and customisable functionalities based on TCGA and GTEx data. The database comprises 9,736 tumours and 8,587 normal control samples (http://gepia.cancer-pku.cn/index.html) [21]. The GEPIA database was used to analyse the transcription level of the membrane androgen and progesterone receptors in ovarian serous cystadenocarcinoma. Ovarian serous adenocarcinoma, the cancer studied by TCGA, is a type of EOC that accounts for about 90% of all OCs.

### 2.4 Kaplan–Meier (KM) analysis

The KM plotter was used to estimate the impact of selected genes encoding membrane progesterone and androgen receptors on survival rates (http://www.kmplot.com/) [22]. KM plotter database assessed the correlation between expression of 54,000 genes and survival in patients with 21 cancer types based on gene arrays, RNA sequences, or next-generation sequencing (for mutation data). Source databases include the GEO, EGA, and TCGA. The correlation between the expression of mRNA encoding the target gene (OXER1 for probe id 222445_at; PGMRC1 for probe id 201121_at; PAQR4 for probe id 212858_at; PAQR7 for probe id 242123_at; PAQR8 for probe id 227626_at) and progression-free survival (PFS), overall survival (OS), and post-progression survival (PPS) in the OC groups was calculated using the KM curve and analysed using the log-rank test. Results are presented as KM survival plots.

### 2.5 Western blot

Expression of PGRMC1 protein in HGrC1, COV434, and SK-OV-3 cells was measured by Western blot analysis. The total protein content of each cell lysate was quantified using a BCA protein assay kit (ThermoFisher Scientific). Proteins were separated by SDS-PAGE and transferred to a PDVF membrane. The membrane was incubated overnight at 4°C with antibody specific for PGRMC1 (#PA5-82040; Invitrogen), followed by horseradish peroxidase (HRP)-conjugated secondary antibody (anti-rabbit [#7074] antibody; Cell Signalling Technology) for 1 h at room temperature. β-Actin (A5316, Sigma-Aldrich) was used as a loading control. Specific protein bands were visualised using WesternBright Sirius Western blotting HRP substrate (Advansta, Menlo Park, CA, USA). Protein bands from independent experiments were then quantified by densitometry using Vision Works LS Acquisition and Analysis software (UVP, Upland, CA, USA).

### 2.6 Viability assay

HGrC1 and COV434 cells were exposed to AG-205 (0.1, 1, 10, 50, and 100 μM; cat# 6242, Bio-Techne Minneapolis, MN, USA) or vehicle (0.1% DMSO) for 24 and 48 h. Cell viability was measured using PrestoBlue Cell Viability Reagent (Invitrogen, Paisley, UK), according to the manufacturer’s instructions. Briefly, the PrestoBlue stock solution was aseptically added to the wells at an amount equal to 10% of the culture medium volume. Culture medium alone was used as a blank. Reduction of resazurin to resorufin was determined after incubation for 10 min by measuring fluorescence with an excitation wavelength of 530 nm and an emission wavelength of 590 nm using a Varioskan™ LUX multimode microplate (ThermoFisher Scientific). Data were analysed using SkanIt RE 6.1.1. software (ThermoFisher Scientific). The cells were then examined under a bright-field microscope (Axiocam 503; 20× objective; Zeiss, Oberkochen, Germany).

### 2.7 Statistical analysis

qPCR, Western blot, and viability data are presented as the mean ± standard error of the mean of three independent experiments, each performed in triplicate. One-way analysis of variance or the Kruskal–Wallis nonparametric equivalent test was used to calculate *p*-values. A *p*-value was considered significant at *p ≤ 0.05, **p ≤ 0.01, ***p ≤ 0.001, and ****p ≤ 0.0001. Data from GEPIA were analysed using Student’s *t* test, and a *p*-value of <0.05 was deemed significant. The hazard ratio (HR) and 95% confidence interval from KM plot analysis were calculated automatically by the website tool. The values for each group are expressed as the mean  ±  SD.

## 3 Results

### 3.1 Expression of ZIP9 (*SLC39A9*)

**Figure 2:**
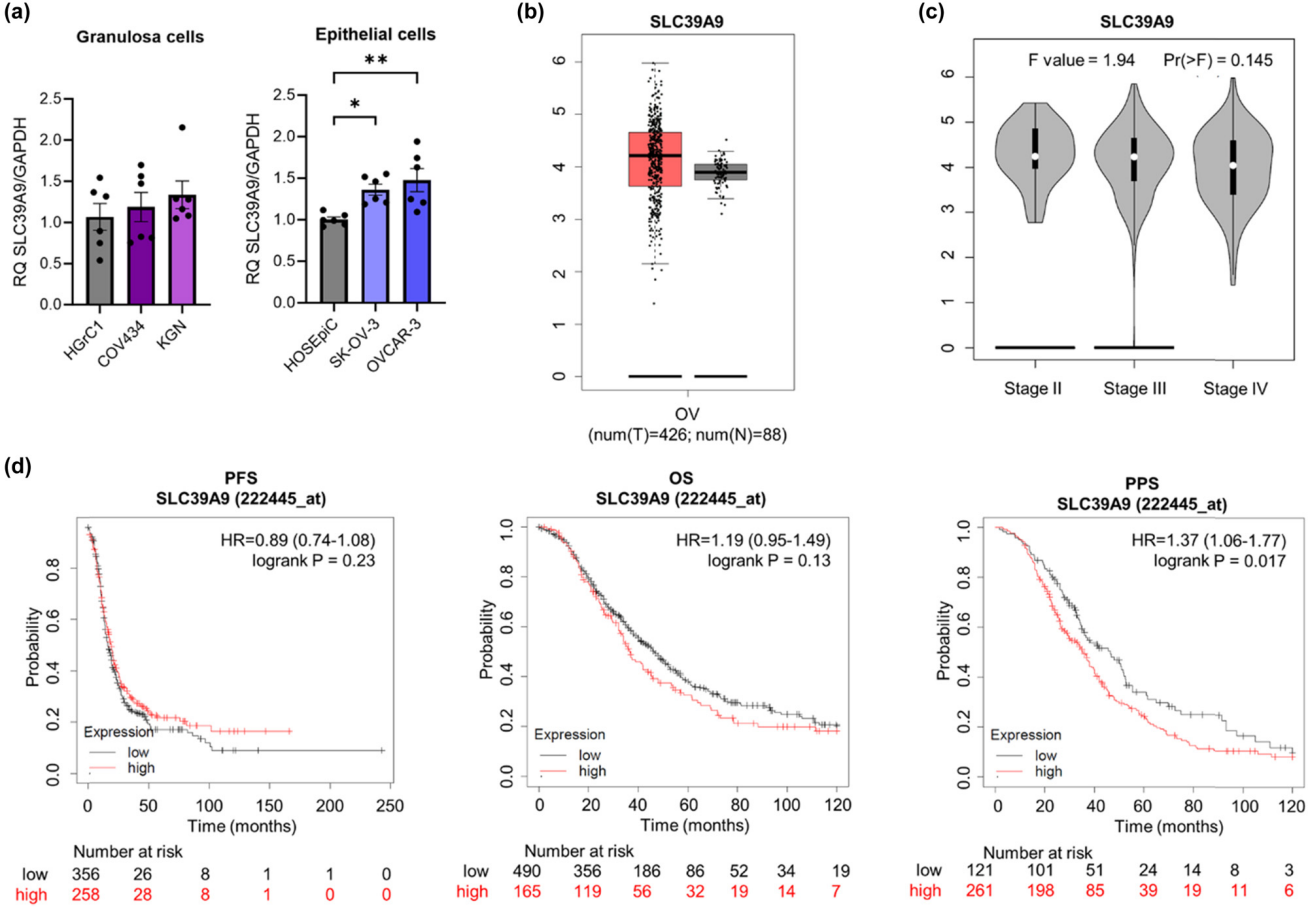
Comparison of ZIP9 mRNA levels in (a) epithelial (HOSEpiC, SK-OV-3, OVCAR-3) and granulosa (HGrC1, COV434, KGN) ovarian normal and cancer cell lines, and (b) in ovarian cancer and normal ovarian tissues (based on analysis of open-source data from the TCGA and GTEx databases using the GEPIA online tool). (c) Correlation between ZIP9 expression and tumour stage in patients with ovarian cancer patients. (d) KM survival plots based on ZIP9 expression, showing PFS, OS, and PPS of ovarian cancer patients with different tumour expression of ZIP9. The HR is indicated, along with the 95% confidence interval in brackets. *p ≤ 0.05 and **p ≤ 0.01.

To assess the expression profile of ZIP9 during OC progression, we first performed RT-qPCR using human epithelial and granulosa OC cell line panels, which also contained normal cell lines for comparison. The results revealed that expression of *ZIP9* mRNA in EOC OVCAR-3 and SK-OV-3 cells was higher than that in HOSEpiC normal epithelial cells (Figure 2a; *p* ≤ 0.01 and *p* ≤ 0.05, respectively), whereas that in granulosa OC cells was comparable with that in normal ovarian cell lines (Figure 2a). We then used the GEPIA database to check the expression profiles of tumour and normal tissues derived from patients (Figure 2b). There were no statistically significant differences between normal and tumour tissues. Then, we used the same database to check whether the high variability in *ZIP9* expression by OC is related to the tumour stage. The results showed no differences in *ZIP9* expression at different tumour stages (Figure 2c). We then analysed KM plots (PFS, OS, and PPS) and found no correlation between the expression of the ZIP9 gene and the survival time of OC patients (Figure 2d). To check whether the ZIP9 gene is essential for OC cells, we performed a Cancer Dependency Map (DepMap) analysis of the gene effect score based on CRISPR knockout screens from Broad’s Achilles and Sanger’s SCORE projects. This analysis revealed negative scores for most of the available OC cell lines, which implies cell growth inhibition and/or death following knockout of the ZIP9 gene; however, the scores are above –0.5, which suggests a rather weak dependency (Figure S1).

### 3.2 Expression of OXER1 (*OXER1*)

**Figure 3:**
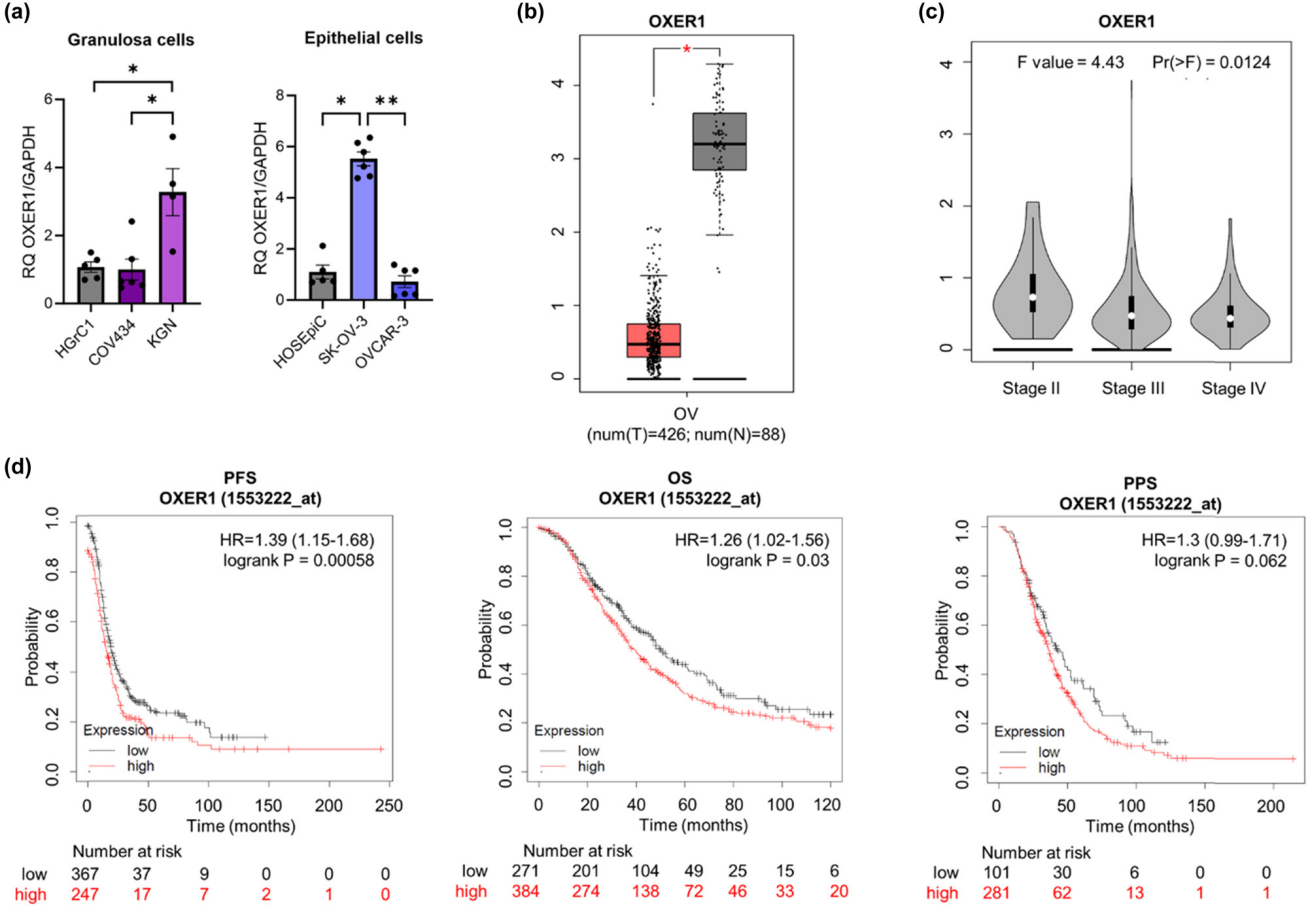
Comparison of OXER1 mRNA levels in (a) epithelial (HOSEpiC, SK-OV-3, OVCAR-3) and granulosa (HGrC1, COV434, KGN) ovarian normal and cancer cells and (b) ovarian cancer and normal ovarian tissue (based on analysis of open source from the TCGA and GTEx mRNA databases using the GEPIA online tool). (c) Correlation between expression of OXER1 and tumour stage of OC patients and (d) KM survival curves related to OXER1 expression, showing PFS, OS, and PPS of OC patients with different tumour expression of OXER1. The HR is indicated, along with the 95% confidence interval in brackets. *p ≤ 0.05 and **p ≤ 0.01.

RT-qPCR to detect OXER1 mRNA revealed higher expression in the rare EOC cell line (SK-OV-3) and in granulosa tumour cells (KGN) than in normal epithelial and granulosa cells, respectively (Figure 3a; *p* ≤ 0.05 and *p* ≤ 0.05). By contrast, expression levels of OXER1 mRNA in the most common EOC (represented here by OVCAR-3 cells) were similar to that in non-cancer epithelial cells (HOSEpiC) (Figure 3a). However, the GEPIA database showed that tumours express markedly lower levels of OXER1 mRNA than normal tissues (Figure 3b, *p* < 0.05), and that expression decreases as the cancer stage increases (Figure 3c). Interestingly, KM plot analysis revealed that in almost all analysed groups, patients with low OXER1 expression survived for longer (PFS and OS) than those with high OXER1 expression (Figure 3d). DepMap analysis (see Supplementary materials) showed positive effect scores for most available OC cell lines, suggesting that OXER1 KO is not associated with cell growth inhibition and/or death (Figure S2).

### 3.3 Expression of PGRMC1 (*PGRMC1*)

**Figure 4:**
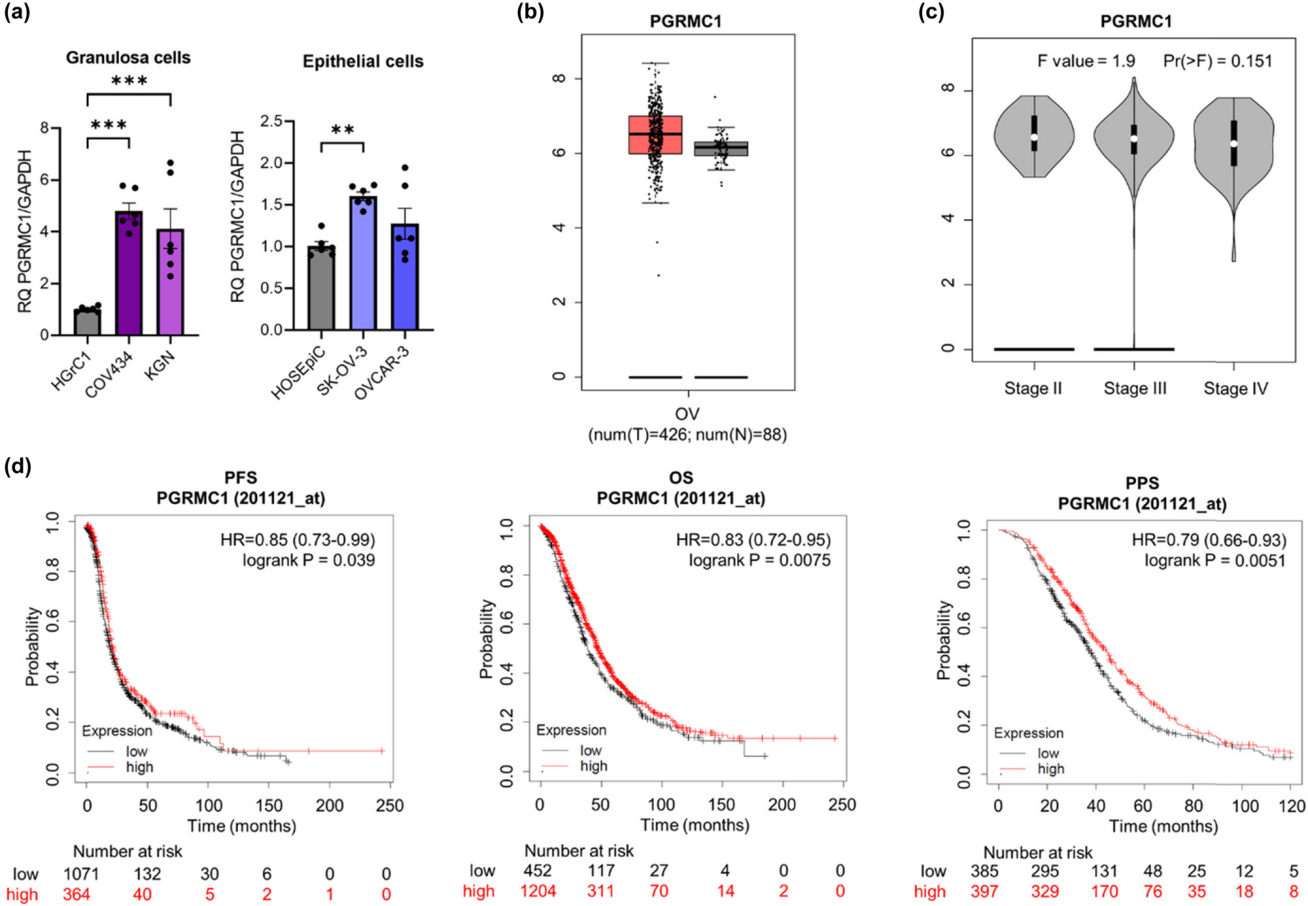
Comparison of PGRMC1 mRNA levels in (a) epithelial (HOSEpiC, SK-OV-3, OVCAR-3) and granulosa (HGrC1, COV434, KGN) ovarian normal and cancer cell lines and (b) ovarian cancer and normal ovarian tissues (based on analysis of open-source data from the TCGA and GTEx mRNA data using the GEPIA online tool). (c) The correlation between PGRMC1 expression and OC stage. (d) KM survival curves related to PGRMC1 expression, showing PFS, OS, and PPS of OC patients with different tumour expression of PGRMC1. The HR is indicated, along with the 95% confidence interval in brackets. *p ≤ 0.05, **p ≤ 0.01, and ***p ≤ 0.001.

RT-qPCR of PGRMC1 mRNA (Figure 4a) showed significantly higher expression in all tested human granulosa OC cell lines than in normal cells (*p* ≤ 0.001). Similar results were obtained for SK-OV-3 (*p* ≤ 0.01). However, expression by OVCAR-3 (the most common EOC) cells was not different from that in normal HOSEpiC cells (Figure 4a). This expression profile was consistent with GEPIA of cancer tissue from patients with EOC (Figure 4b). At the same time, we did not find any stage-dependent changes in patient-derived tumours (Figure 4c). We also noted better survival rates for patients with ovarian serous cystadenocarcinoma showing high PGRMC1 expression (Figure 4d). Gene effect scores calculated from DepMap analysis revealed a weak dependency of most available OC cell lines on expression of the PGRMC1 gene, resulting in a negative score (Figure S3).

### 3.4 Expression of PAQR4 (*PAQR4*)

**Figure 5:**
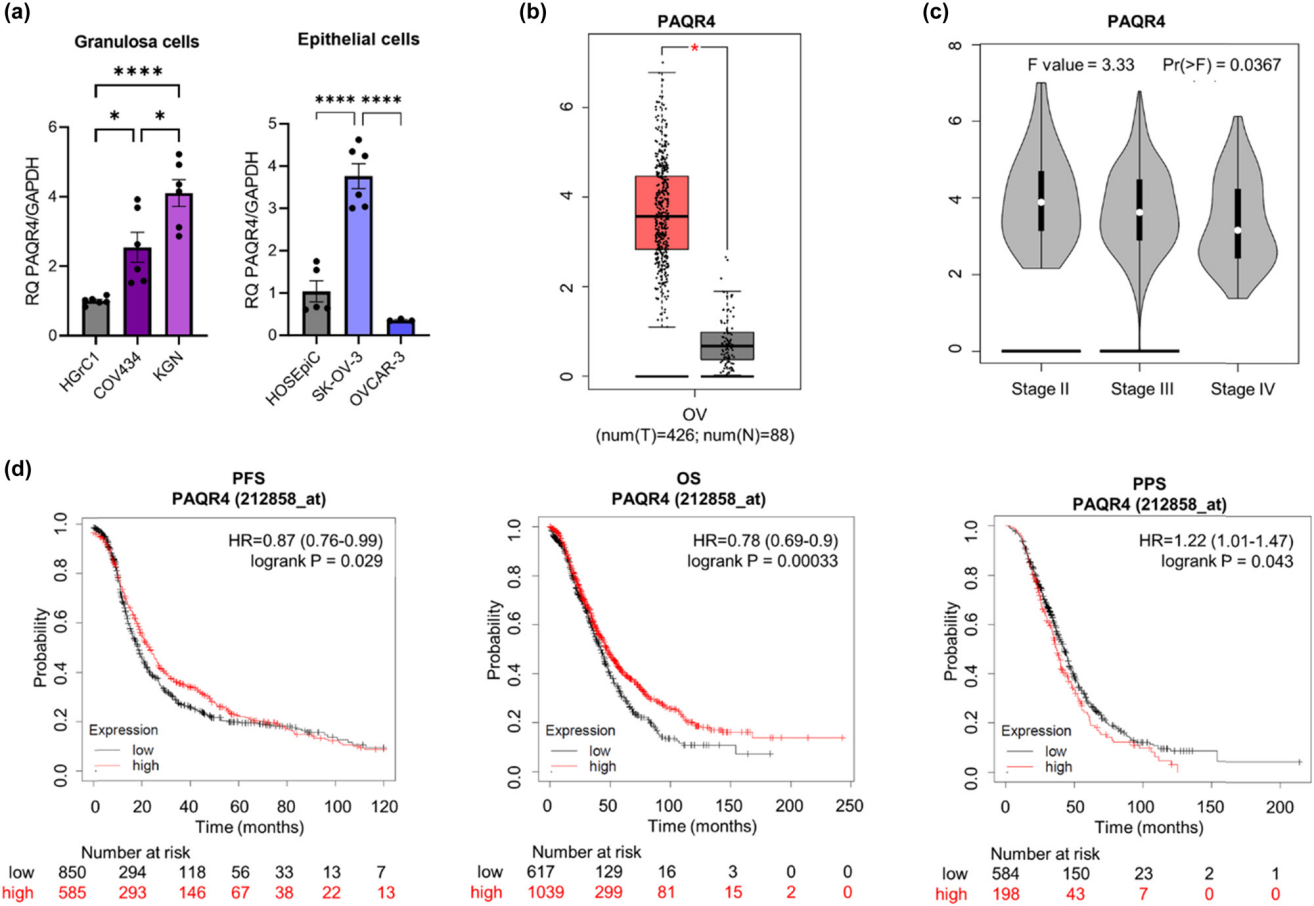
Comparison of PAQR4 mRNA levels in (a) epithelial (HOSEpiC, SK-OV-3, OVCAR-3) and granulosa (HGrC1, COV434, KGN) ovarian normal and cancer cell lines and (b) ovarian cancer and normal ovarian tissues (based on analysis of open-source data from the TCGA and GTEx mRNA databases using the GEPIA online tool). (c) The correlation between PAQR4 expression and stage of OC. (d) KM survival curves related to PAQR4 expression, showing PFS, OS, and PPS of OC patients with different tumour expression of PAQR4. The HR is indicated, along with the 95% confidence interval in brackets. *p ≤ 0.05 and ****p ≤ 0.0001.

Our analysis of PAQR4 expression revealed a marked increase in mRNA levels in all tested rare OC-like granulosa cell lines, as well as in the low-grade EOC line SK-OV-3, compared with normal cells (Figure 5a; *p* < 0.05 for COV434; *p* < 0.0001 for KGN; and *p* < 0.0001 for SK-OV-3). These results are consistent with data from patients in the GEPIA, which show significantly higher expression in cancer tissues than in normal tissue (Figure 5b, *p* < 0.05). By contrast, GEPIA data for tissues derived from three different stages of OC show that expression of this gene decreases with increasing cancer stage (Figure 5c); however, expression of PAQR4 in OC tissues was highly variable. Moreover, KM plot analysis (Figure 5d) showed that patients with ovarian serous cystadenocarcinoma expressing high levels of PAQR4 had longer PFS and OS. There were no differences in PAQR4 mRNA levels between OVCAR-3 cells and HOSEpiC cells (Figure 5a). The gene effect score from DepMap analysis was negative (above –0.5) for most available OC cell lines, suggesting that PAQR4 gene knockout is only weakly associated with cell growth inhibition and/or death (Figure S4).

### 3.5 Expression of PAQR7 (*PAQR7*)

**Figure 6:**
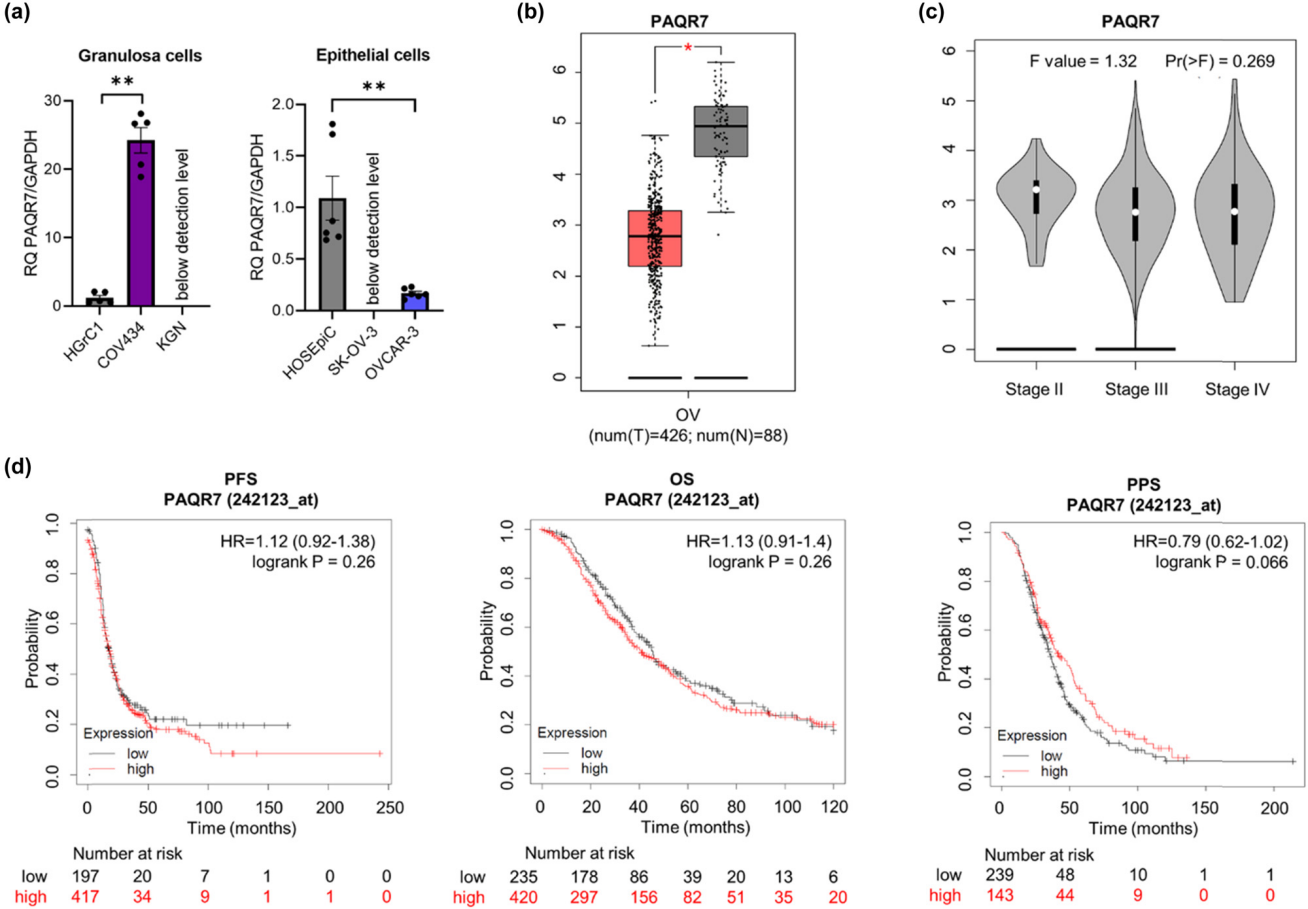
Comparison of PAQR7 mRNA levels in (a) epithelial (HOSEpiC, SK-OV-3, OVCAR-3) and granulosa (HGrC1, COV434, KGN) ovarian normal and cancer cell lines and (b) in ovarian cancer and normal ovarian tissues (based on analysis of open-source data from the TCGA and GTEx mRNA database using the GEPIA online tool). (c) The correlation between PAQR7 expression and tumour stage in OC patients. (d) KM survival curves related to PAQR7 expression, showing PFS, OS, and PPS of OC patients with different tumour expression of PAQR7. The HR is indicated, with the 95% confidence interval in brackets. *p ≤ 0.05 and **p ≤ 0.01.

RT-qPCR revealed significantly lower expression of PAQR7 mRNA in OVCAR-3 EOC than in normal OC (*p* ≤ 0.01), whereas expression in SK-OV-3 and KGN cells was below the limit of detection (Figure 6a). These results are consistent with GEPIA data for patient cancer tissues (Figure 6b; *p* ≤ 0.05). However, expression was much higher in COV434 granulosa cancer cells than in HGrC1 normal granulosa cells (Figure 6a; *p* ≤ 0.01). Moreover, expression of PAQR7 mRNA did not correlate with tumour stage (Figure 6c) or with survival rates (Figure 6d). The DepMap analysis (Supplementary materials) revealed negative scores (above –0.5) for most available OC cell lines, suggesting that PAQR7 knockout is only weakly associated with cell growth inhibition and/or death (Figure S5).

### 3.6 Expression of PAQR8 (*PAQR8*)

**Figure 7:**
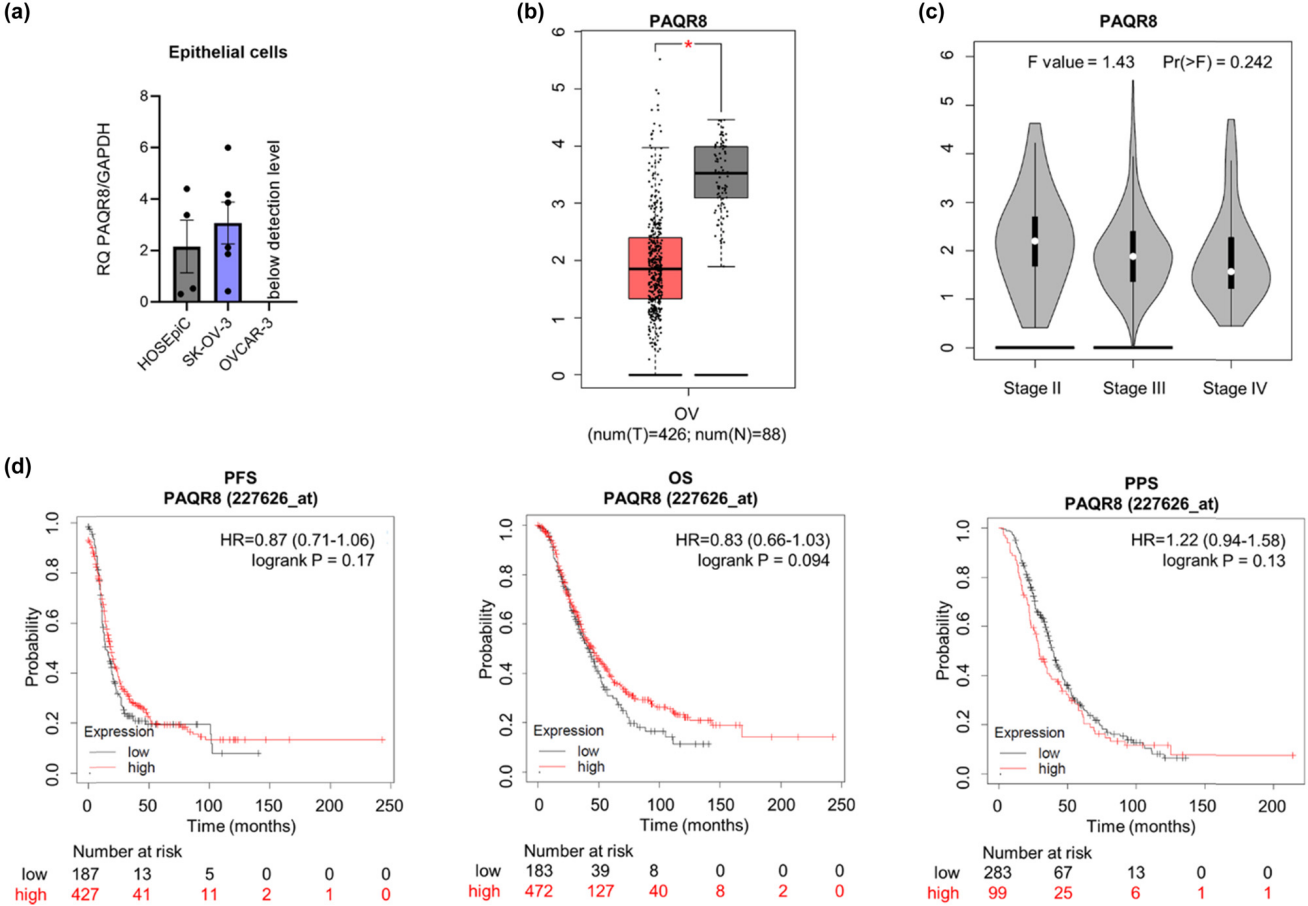
Comparison of PAQR8 mRNA levels in (a) epithelial (HOSEpiC, SK-OV-3, OVCAR-3) ovarian normal and cancer cell lines and (b) ovarian cancer and normal ovarian tissues (based on analysis of open-source TCGA and GTEx mRNA data using the GEPIA online tool). (c) The correlation between expression of PAQR8 and stages of OC. (d) KM survival curves related to PAQR8 expression, showing PFS, OS, and PPS of ovarian cancer patients with different tumour expression of PAQR8. The HR is indicated, with the 95% confidence interval in brackets. *p ≤ 0.05.

RT-qPCR data showed that for most cell lines in our panels, PAQR8 expression was below the limit of detection. Expression was detected only in epithelial SK-OV-3 and HOSEpiC cells (the levels were similar) (Figure 7a). An analysis of PAQR8 transcript levels in the GEPIA database revealed that expression of this gene in OC tissue is significantly lower than in normal tissue (Figure 7b; *p* ≤ 0.05); however, expression was not related to the tumour stage (Figure 7c) or survival rates (Figure 7d). DepMap analysis revealed negative scores (above –0.5) for most available OC cell lines, which implies that knocking out the PAQR8 gene is associated only weakly with cell growth inhibition and/or death (Figure S6).

### 3.7 Rare OC: PGRMC1 protein expression and viability analysis

**Figure 8:**
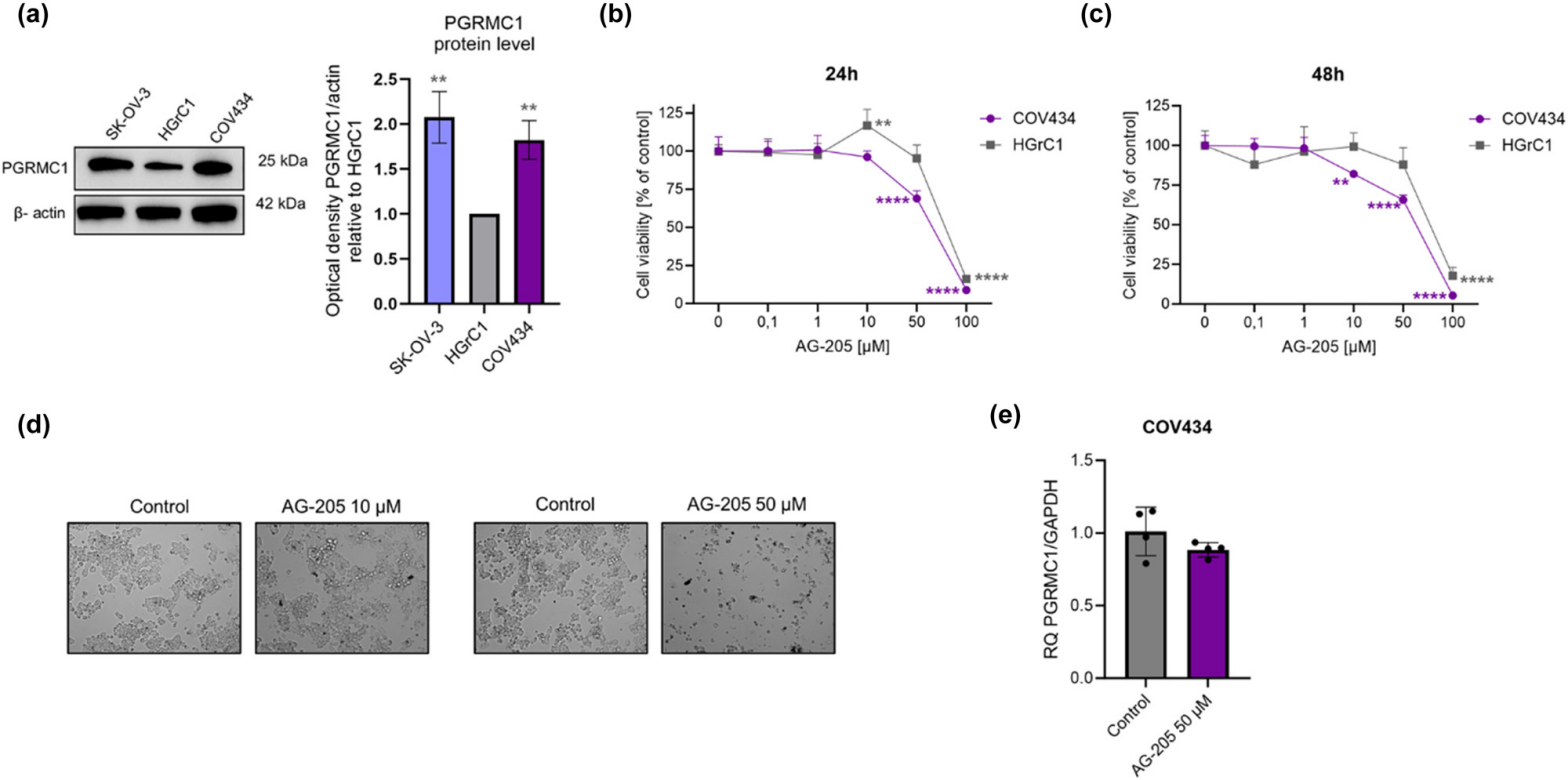
AG-205 reduces cell viability in PGRMC1-positive cells. (a) Comparison of PGRMC1 protein level in rare OC (SK-OV-3, COV434) and non-cancer (HGrC1) cells. Dose-dependent effect on cell viability in COV434 and HGrC1 cells following 0, 0.1, 1, 10, 50, and 100 μM AG-205 treatment for (b) 24 h and (c) 48 h. (d) A bright-field microscope analysis following 0, 10 and 50 μM AG-205 treatment for 48 h in COV434 cells (20× magnification). (e) PGRMC1 mRNA expression following 50 μM AG-205 treatment in COV434 cells. **p ≤ 0.01 and ****p ≤ 0.0001.

Immunoblotting showed that PGRMC1 was expressed at higher levels in SKOV-3 and COV434 compared to noncancer HGrC1 cells (Figure 8a; *p* ≤ 0.05). To further elucidate the role of PGRMC1 in rare OC, we treated COV434 cells with the PGRMC1 ligand AG-205. In our experiments, we used AG-205 at concentrations at or below the concentrations known to inhibit PGRMC1 activity [23]. AG-205 reduced the viability of COV434 in a dose-dependent manner, while showing minimal effects on non-cancer cells (Figure 8b–d; *p* ≤ 0.01, *p* ≤ 0.0001), except at the high dose which was toxic to both cell types. Analysis of bright field images showed that control cells were flattened and had all the characteristics of normal, healthy cells (Figure 8d). However, cells treated with AG-205 showed changes in morphology, poor attachment to the surface, and lack of characteristic cellular projections (Figure 8d). In addition, AG-205 at 50 μM did not alter PGRMC1 gene expression levels in the COV434 cell line (Figure 8e).

## 4 Discussion

OC is a malignancy that depends on binding of hormones, particularly steroid hormones, to their cognate receptors to drive growth. Here, we investigated expression of transcripts for different membrane-bound receptors for androgens and progesterone in different subtypes of human OC cell lines panel. We found that although these receptors are expressed by many subtypes of OC cell lines, as well as in patient tissues, expression patterns differ markedly according to subtype.

### 4.1 Membrane androgen receptors

Androgen signalling promotes proliferation, migration, and invasion of OC cells [24]. ZIP9 and OXER1 are plasma membrane receptors that mediate the activities of androgens [25,26]; however, to the best of our knowledge, the role of ZIP9 and OXER1, and their expression levels in different subtypes of OC, is largely unknown.

ZIP9 (also known as Zrt- and Irt-like Protein 9 and Solute Carrier family 39 member 9, SLC39A9) is a Zn^2+^ transporter protein that regulates Zn^2+^ influx from the extracellular space to the cytoplasm [27]; it also functions as a mAR that is coupled to G proteins [28]. The current study revealed high variability in the expression of *ZIP9* between OC tissues from patients. Interestingly, our studies showed that the expression of *ZIP9* was upregulated in EOC subtypes, with a significance level of *p* ≤ 0.01 for OVCAR-3 and *p* ≤ 0.05 for SK-OV-3, while no changes were observed in GCT lines. Overexpression of *ZIP9* has been reported in breast cancer tissues compared with normal breast tissues [29]. Moreover, overexpression of unliganded ZIP9 in breast cancer cells (MDA-MB-231 cells) increases zinc levels and promotes cell migration/invasion [30]. Despite this, we did not find any correlation between the expression of the ZIP9 gene and tumour stage/survival time in OC patients. In summary, ZIP9 is widely expressed in OC subtypes but does not seem to have prognostic value for OC.

OXER1 is a G protein-coupled receptor (GPCR; previously known as G Protein-coupled Receptor 170 [GPR170], hGPCR48, HGPCR48, TG1019, or R527) [31]. Here, analysis of GEPIA data suggested lower expression of OXER1 in OC tissue than in normal ovarian tissue, which correlates positively with patient survival; however, the results for other subtypes of OC showed the opposite. Thus, we conclude that expression of OXER1 is higher in SK-OV-3 (*p* ≤ 0.05) and KGN (*p* ≤ 0.05) cell lines than in normal cells, whereas that in OVCAR-3 and COV434 is the same as that in normal cells. Previous reports suggested a similar pattern for the nuclear androgen receptor in different subtypes of OC [32–34]; therefore, we suggest that OXER1 has no utility as an independent prognostic marker for OC.

### 4.2 Membrane progesterone receptor

The results of studies on the effect of progesterone in OC are inconsistent, with both proliferative and inhibitory activity being reported [35–38]. Membrane progesterone receptors are classified into two groups: mPRs and MAPRs [39].

The mPRs are 7-transmembrane protein receptors located in the cell plasma membrane; their function is to transduce signals via G proteins [40]. The human genome encodes 11 members (PAQR1 to PAQR11). The prototypes in this family are PAQR5–8, which function as membrane receptors for progesterone [41]. The results of this study show that PAQR7 (mPRα) and PAQR8 (mPRβ) were downregulated significantly in OC patients; indeed, expression in most of the cell lines in our panels was below the limit of detection. PAQR7 plays an important role in regulating a wide range of progestin-induced effects, including progestin-induced oocyte maturation, ovarian follicle growth, and onset of parturition [42]. Previous research showed that PAQR7 is expressed in normal reproductive tissues, including the ovary [42], which we also observed in our panel of OC cell lines. We found that both human ovarian epithelial HOSEpiC and granulosa HGrC1 cells express the PAQR7 receptor. In addition, we found that in OVCAR-3 cells and patient tissues, expression of PAQR7 was lower in cancer cells than in normal cells, with a significance level of *p* ≤ 0.01 and *p* ≤ 0.05, respectively. Moreover, expression in SK-OV-3 and KGN cells was below the limit of detection. Interestingly, we found that the transcript level for the PAQR8 receptor was below the detection limit in normal and cancerous granulosa cells. Furthermore, we only detected the expression of this receptor in normal epithelial cells (HOSEpiC) and rare EOC SK-OV-3 cells. These observations are consistent, at least in part, with previously published studies showing expression of PAQR7 and PAQR8 in both SK-OV-3 and OVCAR-3 cells [43] and in tissue samples of common types of OC [44]; however, we found that expression of both PAQR7 and PAQR8 was low or undetectable in almost all analysed OC cell lines, regardless of rare or common type, indicating that these receptors may have prognostic value, or may be a target for treatment in the future.

Since PAQR4 was identified recently as a novel tumour suppressor [45], we analysed its expression, even though it is not a recognised progesterone receptor. PAQR4 protein plays a major role in cancer cell proliferation, migration, invasion, and epithelial-mesenchymal transition, as well as in the suppression of apoptosis [46]. We found that PAQR4 was overexpressed in all rare OC cells (*p* ≤ 0.05 for COV434; *p* ≤ 0.0001 for KGN and SK-OV-3) and patient tissues but not in OVCAR-3 cells. A recent study showed that PAQR4 is expressed at higher levels in the tumour tissue of patients with breast cancer than in corresponding normal tissues and that its expression correlates negatively with patient survival [47]. Also, the knockdown of PAQR4 in human breast cancer cell lines SUM159 and MCF7 suppressed cell proliferation [38]. Similarly, Xu et al. showed that knockdown of PAQR4 inhibits proliferation in non-small-cell lung cancer cells, mainly by inducing cellular apoptosis both *in vivo* and *in vitro* [45]. Furthermore, depleting PAQR4 increases the sensitivity of these cancer cells to chemotherapy. However, it is unclear whether PAQR4 is involved in OC apoptosis or drug resistance, although this is worthy of further study. We conclude therefore that PAQR4 could be a new target protein for treatment of rare OC.

PGRMC1 is a MAPR with a single transmembrane domain that binds to several ligands in addition to progestins (i.e. cholesterol, glucocorticoids, and other steroids) [48]. This protein takes part in many processes important for cancer, e.g. cell viability, apoptosis, entry into the cell cycle, and subsequent progression of cell division [49]. In our study, we found increased expression of PGRMC1 in rare OC cells, with a significance level of *p* ≤ 0.001 for GCT and *p* ≤ 0.01 for SK-OV-3; however, this was not the case in more common OC subtypes (represented by OVCAR-3 cells), or in the GEPIA database. Unexpectedly, EOC patients with high PGRMC1 expression show better survival rates. Nevertheless, activation of PGRMC1 by progesterone promotes survival of OVCAR-3 cells and makes them more resistant to platinum-based chemotherapy [44]. In this context, overexpression of PGRMC1 in rare OC subtypes and its role in growth and chemoresistance highlight the need for further studies.

To investigate the PGRMC1 signalling mechanism involved in rare OC viability, we used the chemical inhibitor AG-205; this aromatic compound has high affinity binding to PGRMC1 [50] and is able to inhibit PGRMC1 downstream signalling [23,51]. The results of our studies show that disruption of PGRMC1 signalling by AG-205 reduced cell viability in tumour (COV434) and non-tumour (HGrC1) cell lines. However, the effect was stronger in tumour cells with high PGRMC1 expression. Our results are in line with a previous observation that inhibition and silencing of PGRMC1 in breast cancer decreased cell proliferation, migration, and invasiveness, as well as inducing cell-cycle arrest and apoptosis of cancer cells. By contrast, overexpression of PGRMC1 results in increased cell proliferation of cancer cell lines [52]. Interestingly, AG-205 did not decrease PGRMC1 expression in breast cancer cells [52], as in our study. These results suggest that AG-205 acts as an inhibitor of PGRMC1 signalling rather than a gene down regulator. Our observations also suggest that PGRMC1 may be a good target protein for treatment, particularly of rare subtypes of OC showing increased PGRMC1 expression.

The expression profiles of receptors expressed by the OC panel revealed something significant. The receptor expression profile depended on the subtype of OC. In addition, COV434 cells have a different expression profile than KGN cells with respect to almost all receptors, even though both of these cell lines are described as granulosa tumours. These results are consistent with our previous studies suggesting that both cell lines can synthesise oestradiol, despite having different genetic backgrounds [10,53]. In addition, a recent study based on available morphological, immunohistochemical, genetic, and clinical features suggests that COV434 is derived from small cell OC, a very rare subtype of OC [9]. Similarly, we observed genetic differences between the SK-OV-3 and OVCAR-3 cell lines. Both are widely used in OC research, but only OVCAR3 is unambiguously derived from HGSOC, while SK-OV-3 is considered “not HGSOC” [10]. In addition, Beaufort et al. classified the SK-OV-3 line as being derived from clear cell OC [10]. To summarise, the data suggest that both SK-OV3 and COV434 cells need to be re-assigned.

In our work, we used bioinformatics data for patients and our RT-qPCR analysis for cell lines in OC tissues. We analysed the expression of membrane receptors in a very rare subtype of OC. For example, the number of patients with ovarian granulosa tumours in the EU ranges from 33 to 260 per year/country. This has made these results extremely difficult to obtain and replicate in patient tissue. However, our functional analysis shows that disruption of PGRMC1 signalling by AG-205 inhibits cell growth in the rare OC subtype represented by COV434 cells. As these are preliminary studies, we have not been able to perform a similar analysis on a representative group of patients in our laboratory at this stage. This is a limitation of this study at the moment but also a future direction.

In conclusion, until recently, clinical trials have included all subtypes of EOC, leading to a uniform treatment strategy, whereas clinical trials of non-EOC are extremely rare. The present study demonstrates that PGRMC1 and PAQR4 are new and promising potential target proteins for anticancer therapy, particularly for rare subtypes of OC; however, further studies are needed to validate our preliminary data. To extend this study, it would be beneficial to confirm the bioinformatics results in a representative group of patient samples. Another interesting direction will be to confirm the effect of PGRMC1 and PARQ4 gene knockout in rare subtypes of OC cells on proliferation, apoptosis, and cell cycle, as well as on sensitivity to chemotherapy. Furthermore, the development of structure-based compounds tailored to activate or antagonise one or more membrane steroid receptors should be considered a promising tool for the treatment of rare OC subtypes.

## Supplementary Material

Supplementary Figure
