# Supplementary material for: PGRMC1 and PAQR4 are promising molecular targets for a rare subtype of ovarian cancer
Source: Open Life Sci. 2024 Oct 26;19(1):20220982. doi: 10.1515/biol-2022-0982 (PMC11512499; doi:10.1515/biol-2022-0982)
Supplement: Supplementary Figure [file biol-2022-0982-sm.pdf]

Supplementary material

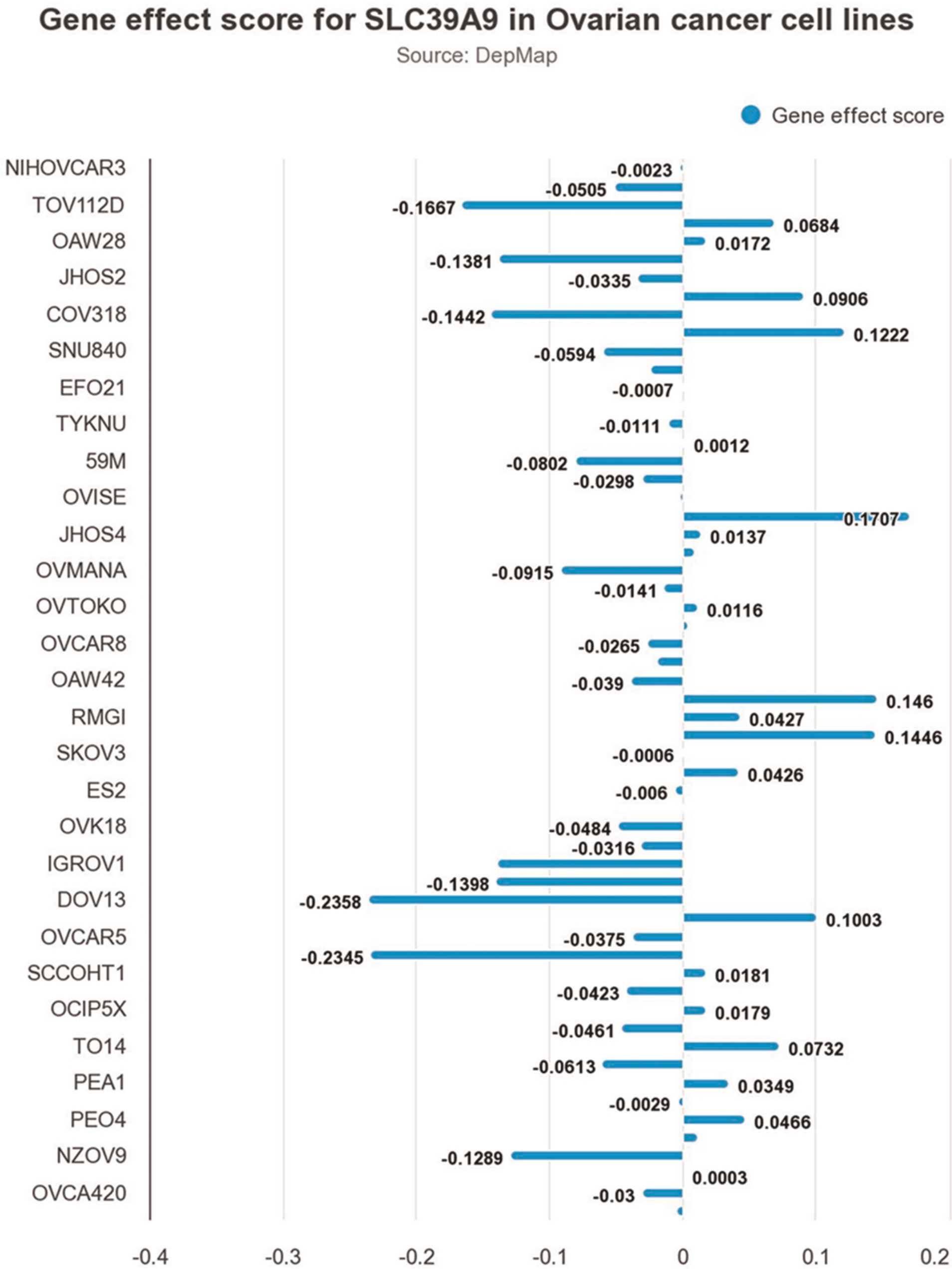

Figure S1: DepMap analysis of gene effect score for ZIP9 in available ovarian cancer cell lines based on data derived from CRISPR knockout screens published by Broad's Achilles and Sanger's SCORE projects. Negative scores imply cell growth inhibition and/or death following gene knockout. Scores are normalized such that nonessential genes have a median score of 0 and independently identified common essentials have a median score of −1. Gene Effect scores were inferenced by Chronos [3]. Integration of the Broad and Sanger datasets was performed as described in Pacini et al. [4], except that quantile normalization was not performed.

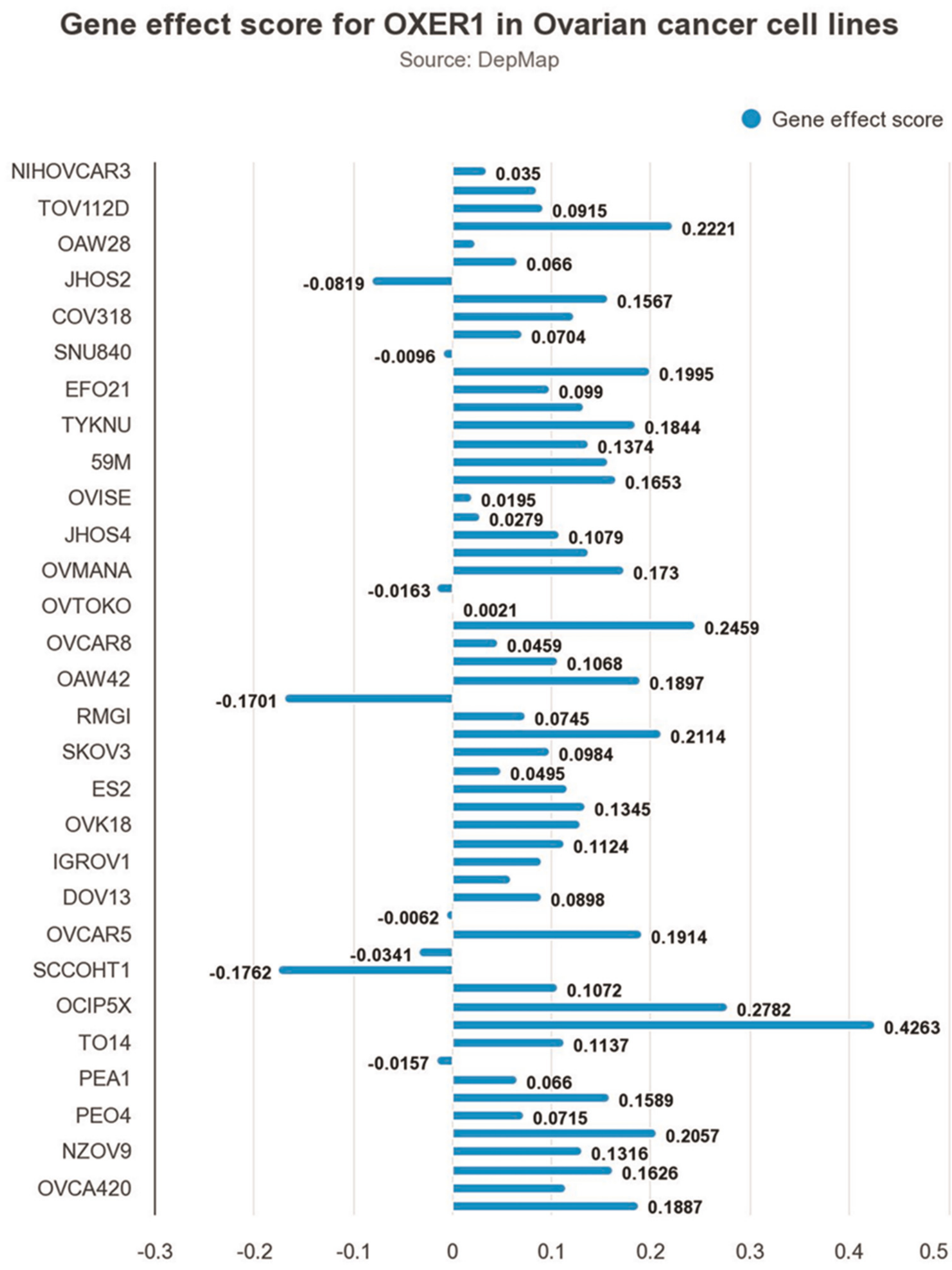

Figure S2: DepMap analysis of gene effect score for OXER1 in available ovarian cancer cell lines based on data derived from CRISPR knockout screens published by Broad's Achilles and Sanger's SCORE projects. Negative scores imply cell growth inhibition and/or death following gene knockout. Scores are normalized such that nonessential genes have a median score of 0 and independently identified common essentials have a median score of −1. Gene Effect scores were inferenced by Chronos [1]. Integration of the Broad and Sanger datasets was performed as described in Pacini et al, 2021 [2], except that quantile normalization was not performed.

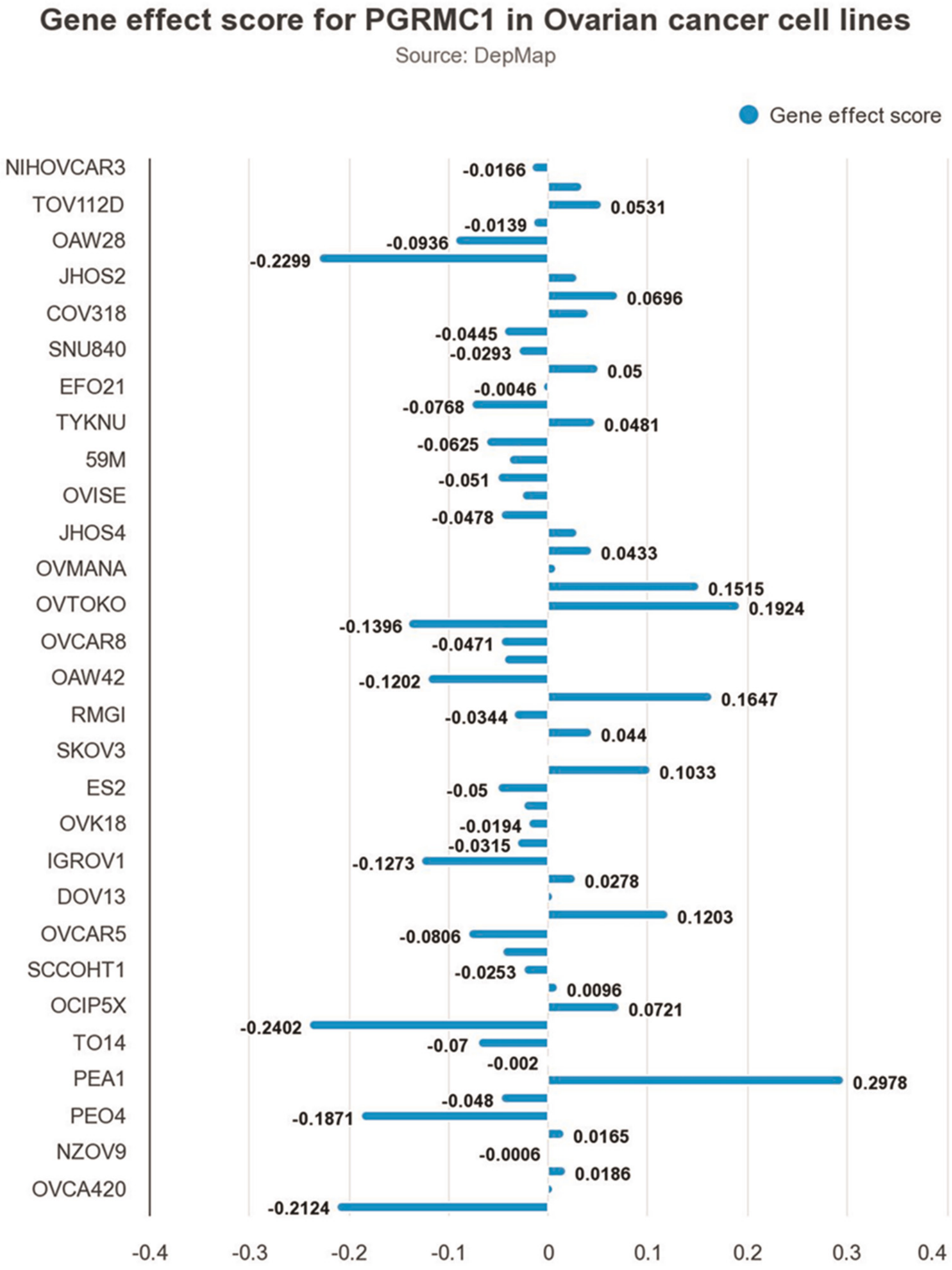

Figure S3: DepMap analysis of gene effect score for PGRMC1 in available ovarian cancer cell lines based on data derived from CRISPR knockout screens published by Broad's Achilles and Sanger's SCORE projects. Negative scores imply cell growth inhibition and/or death following gene knockout. Scores are normalized such that nonessential genes have a median score of 0 and independently identified common essentials have a median score of −1. Gene Effect scores were inferenced by Chronos [3]. Integration of the Broad and Sanger datasets was performed as described in Pacini et al, 2021 [4], except that quantile normalization was not performed.

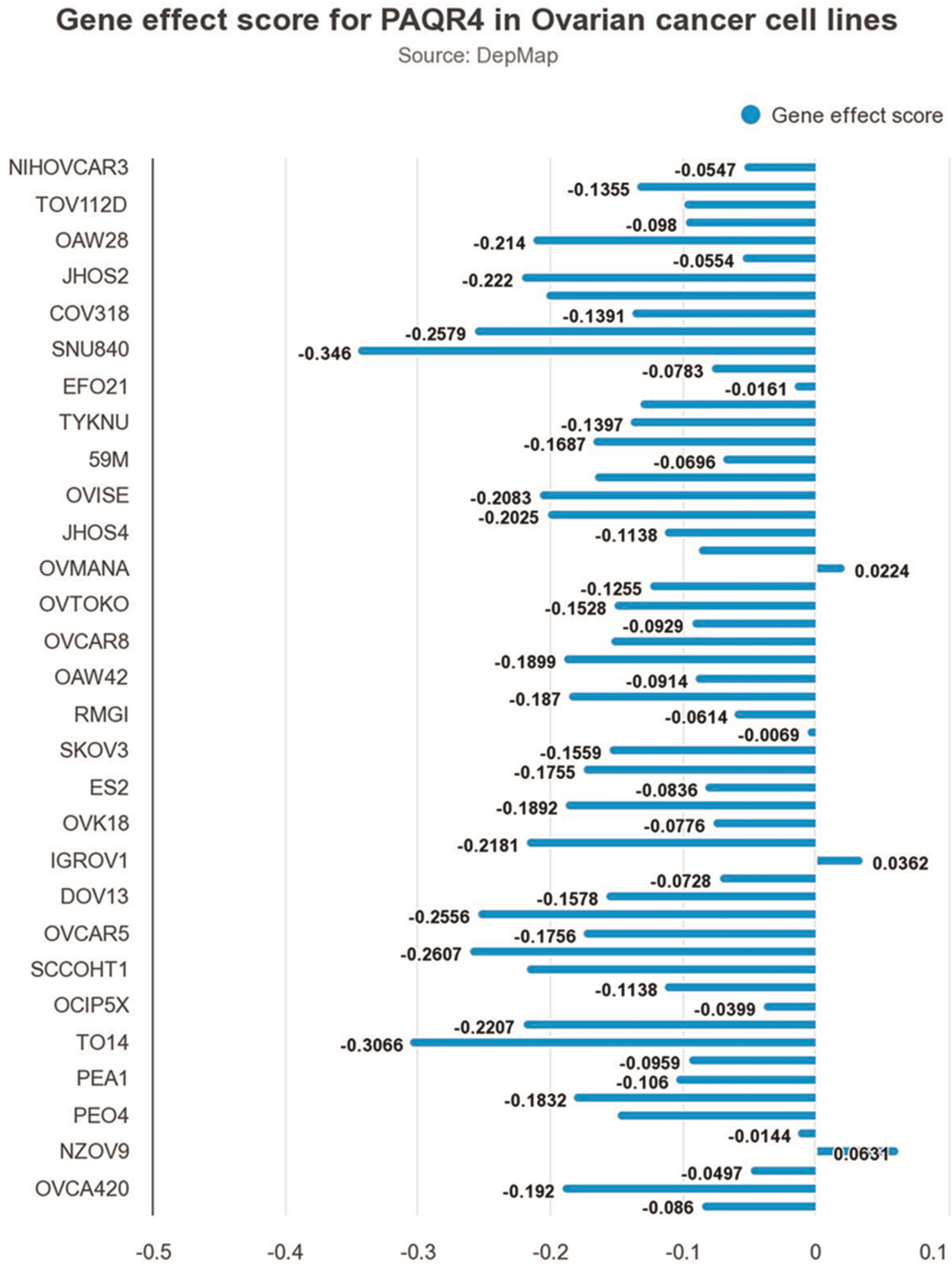

Figure S4: DepMap analysis of gene effect score for PAQR4 in available ovarian cancer cell lines based on data derived from CRISPR knockout screens published by Broad's Achilles and Sanger's SCORE projects. Negative scores imply cell growth inhibition and/or death following gene knockout. Scores are normalized such that nonessential genes have a median score of 0 and independently identified common essentials have a median score of −1. Gene Effect scores were inferenced by Chronos [3]. Integration of the Broad and Sanger datasets was performed as described in Pacini et al. [4], except that quantile normalization was not performed.

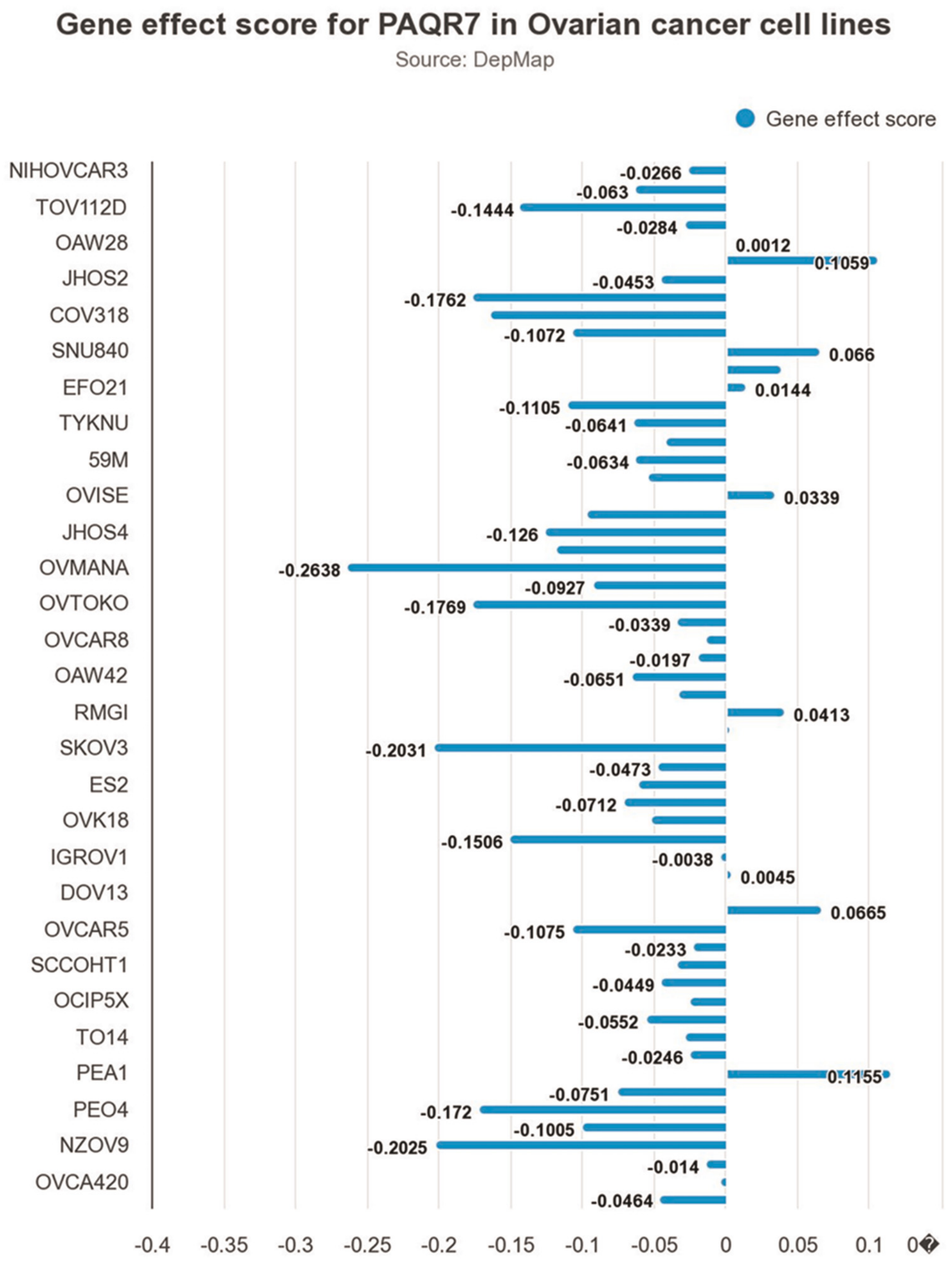

Figure S5: DepMap analysis of gene effect score for PAQR7 in available ovarian cancer cell lines based on data derived from CRISPR knockout screens published by Broad's Achilles and Sanger's SCORE projects. Negative scores imply cell growth inhibition and/or death following gene knockout. Scores are normalized such that nonessential genes have a median score of 0 and independently identified common essentials have a median score of −1. Gene Effect scores were inferenced by Chronos [3]. Integration of the Broad and Sanger datasets was performed as described in Pacini et al. [4], except that quantile normalization was not performed.

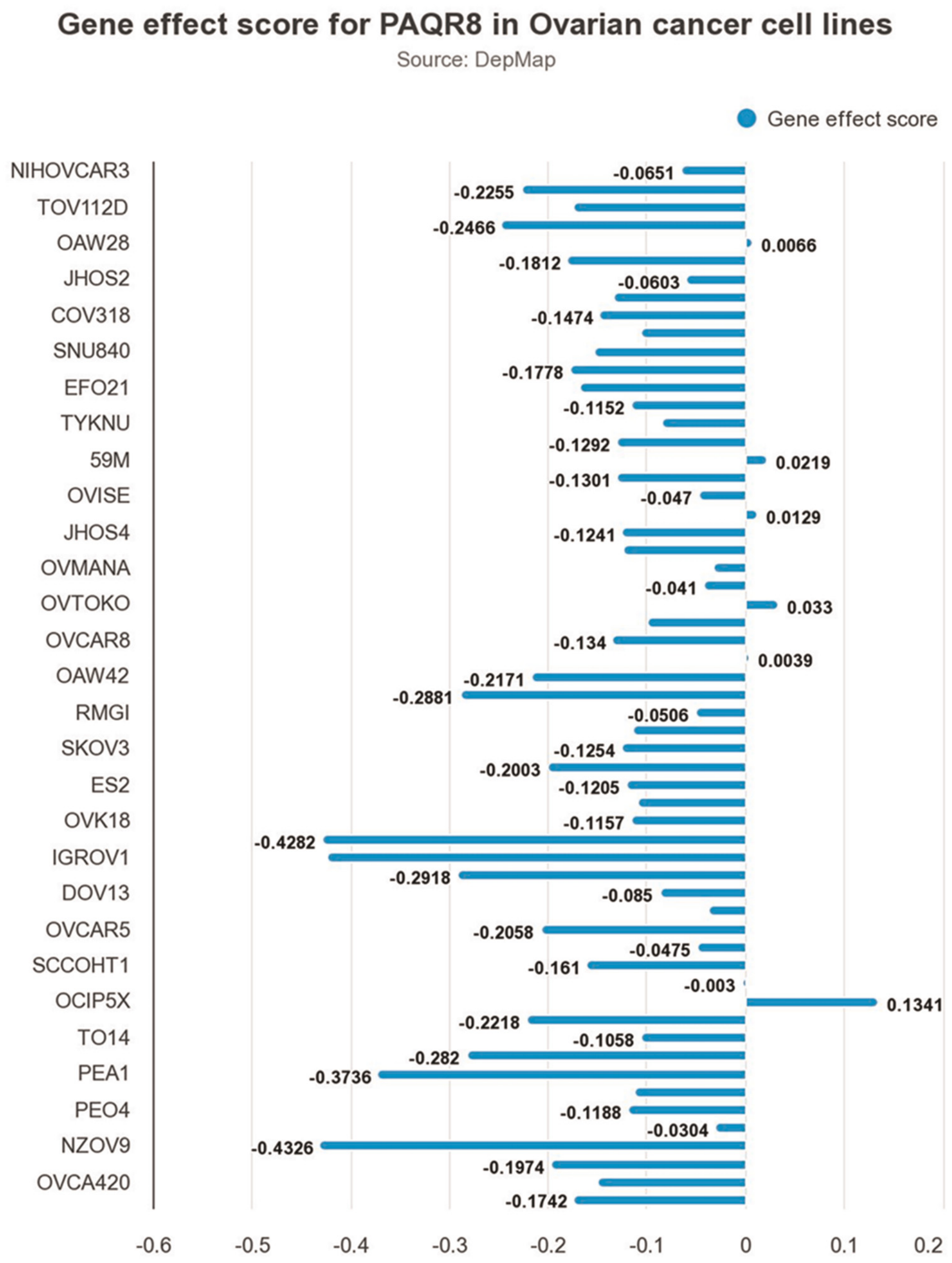

Figure S6: DepMap analysis of gene effect score for PAQR8 in available ovarian cancer cell lines based on data derived from CRISPR knockout screens published by Broad's Achilles and Sanger's SCORE projects. Negative scores imply cell growth inhibition and/or death following gene knockout. Scores are normalized such that nonessential genes have a median score of 0 and independently identified common essentials have a median score of −1. Gene Effect scores were inferenced by Chronos [3]. Integration of the Broad and Sanger datasets was performed as described in Pacini et al. [4], except that quantile normalization was not performed.

References

[1] Gogola J, Hoffmann M, Nimpsz S, Ptak A. Disruption of 17β-estradiol secretion by persistent organic pollutants present in human follicular fluid is dependent on the potential of ovarian granulosa tumor cell lines to metabolize estrogen. Mol Cell Endocrinol. 2020 Mar;503:110698. doi: 10.1016/j.mce.2019.110698. Epub 2019 Dec 28. PMID: 31891770.

[2] Gogola-Mruk J, Hoffmann-Młodzianowska M, Kamińska K, Ptak A. Mixtures of persistent organic pollutants increase ovarian granulosa tumor cell line migration and spheroid invasion by upregulating MMP2 expression and activity via IGF1R. Toxicology. 2021 Mar;452:152715. doi: 10.1016/j.tox.2021.152715. Epub 2021 Feb 8. PMID: 33571556.

[3] Dempster JM, Boyle I, Vazquez F, Root DE, Boehm JS, Hahn WC, et al. Chronos: a cell population dynamics model of CRISPR experiments that improves inference of gene fitness effects. Genome Biol. 2021 Dec;22(1):343. doi: 10.1186/s13059-021-02540-7. PMID: 34930405.

[4] Pacini C, Dempster JM, Boyle I, Gonçalves E, Najgebauer H, Karakoc E, et al. Integrated cross-study datasets of genetic dependencies in cancer. Nat Commun. 2021 Mar;12(1):1661. doi: 10.1038/s41467-021-21898-7. PMID: 33712601.
